# Cross-validation of comorbidity items in two national databases in a sample of patients with end-stage kidney disease

**DOI:** 10.1186/s12913-023-10145-y

**Published:** 2023-10-24

**Authors:** Isabella Vanorio-Vega, Panayotis Constantinou, Assia Hami, Eric Cellarier, Antoine Rachas, Philippe Tuppin, Cécile Couchoud

**Affiliations:** 1 Direction de La Stratégie Des Études Et Des Statistiques, Caisse Nationale de L’assurance Maladie (CNAM), Paris, Cedex 20 75986 France; 2 Agence de La Biomédecine, 1 Avenue du Stade de France, Saint-Denis, 93212 France; 3 Centre Hospitalier Universitaire de Nantes. PHU1-Institut de Transplantation Urologie Néphrologie (ITUN), Centre d’Hemodialyse Chronique- Aile Nord-Zone Administrative RCB, Nantes, France; 4 Centre Hospitalier Universitaire Clermont-Ferrand, Hôpital Gabriel Montpied Département d’Information Médicale, Clermont-Ferrand, 63003 France

**Keywords:** Medico-administrative databases, Algorithm validation, Comorbidities

## Abstract

**Background:**

The use of national medico-administrative databases for epidemiological studies has increased in the last decades. In France, the Healthcare Expenditures and Conditions Mapping (HECM) algorithm has been developed to analyse and monitor the morbidity and economic burden of 58 diseases. We aimed to assess the performance of the HECM in identifying different conditions in patients with end-stage kidney disease (ESKD) using data from the REIN registry (the French National Registry for patients with ESKD).

**Methods:**

We included all patients over 18 years of age who started renal replacement therapy in France in 2018. Five conditions with a similar definition in both databases were included (ESKD, diabetes, human immunodeficiency virus [HIV], coronary insufficiency, and cancer). The performance of each SNDS algorithm was assessed using sensitivity, specificity, positive predictive values (PPVs), negative predictive values (NPVs), and Cohen’s kappa coefficient.

**Results:**

In total 5,971 patients were included. Among them, 81% were identified as having ESKD in both databases. Diabetes was the condition with the best performance, with a sensitivity, specificity, PPV, NPV, and Kappa coefficient all over 80%. Cancer had the lowest level of agreement with a Kappa coefficient of 51% and a high specificity and high NPV (94% and 95%). The conditions for which the definition in the HECM included disease-specific medications performed better in our study.

**Conclusion:**

The HECM showed good to very good concordance with the REIN database information overall, with the exception of cancer. Further validation of the HECM tool in other populations should be performed.

**Supplementary Information:**

The online version contains supplementary material available at 10.1186/s12913-023-10145-y.

## Background

The use of national medico-administrative databases for epidemiological studies has increased in the last two decades as an alternative to traditional observational studies. These databases were conceived to survey healthcare systems from a financial and administrative point of view, with information such as reimbursement claims, healthcare services, medical procedures, daily compensation, etc. [1]. The use of such databases for research has the potential to reduce the risk of selection bias often present in epidemiological surveys, as they are almost exhaustive. In addition, it is less costly, as the data is collected systematically and relatively easily accessible, simultaneously eliminating recall bias, as it relies on data collected systematically and not based on patient reporting with potential recollection mistakes. National databases are helpful for longitudinal studies, as they make it possible to include extended follow-up times and large sample sizes, as well as rare events and epidemiological surveillance or surveys. Such databases are, however, not exempt from information bias [2, 3] as the information tends to be essentially administrative. For example, pharmaceutical information is limited to the dispensation of prescribed and reimbursed medications that are registered in the insurance records [1]. Over the counter medications can be easily missed.

The French population benefits from universal public healthcare coverage. All information concerning the use of the healthcare system is recorded in the National Health Data System (“*Système National des Données de Santé, SNDS*”) [4]. Since 2012, the French National Health Insurance has developed a tool based on the SNDS to analyse and monitor the morbidity and economic burden of 58 treated diseases, chronic treatments, and episodes of care through healthcare utilization [5]. Healthcare Expenditures and Conditions Mapping (HECM) allows the identification of diseases by means of medical algorithms based on the diagnoses for hospitalization, long-term disease diagnoses, and reimbursement of specific treatments for certain diseases for a given year and a period up to four years before. This algorithm is repeated for each year providing a cross-sectional study repeated over time [6]. The HECM has provided information to improve healthcare policies in France (preparing the French Social Security Funding Act and the Public Health Act). The findings of the HECM on disease prevalence and expenditures are similar to those of studies conducted in other countries [6].

A previous study in France compared the performance of various SNDS-based algorithms to identify treated diabetes against clinical data from CONSTANCES (a national French cohort of professionally active or retired salaried workers and their families), showing excellent performance for the three algorithms, including HECM’s current algorithm concerning diabetes [7]. However, such algorithm validity assessments are still scarce. Data from registries offer this opportunity because they provide gold-standard data: they are exhaustive for a given territory, registered manually, and controlled by experienced research assistants.

We aimed to assess the performance of five HECM algorithms on patients with ESKD (ESKD, diabetes, HIV infection, cancer, and coronary disease) against information on the French Renal Epidemiology and Information Network (REIN). The REIN database provides national quality-controlled data on patients with ESKD. It relies on a network of nephrologists, epidemiologists, patients, and public health representatives who are coordinated regionally and nationally by the French biomedical agency, collecting exhaustive information on patients with ESKD (treatment and its changes, demographics, comorbid conditions, treatment center location, etc.) [8, 9].

## Methods

### Data sources

#### The REIN registry

The REIN registry was started in 2002 and covered all of France by 2012. It includes all patients receiving renal replacement therapy (RRT) in mainland France and its overseas territories. The REIN database collects information on patient characteristics (body mass index [BMI], age, sex, RRT modality, date of RRT start) and conditions (e.g., diabetes, coronary artery disease, cancer) based on medical records. Nephrologists, health managers, nurses, medical secretaries, and research assistants collect the data. Continuous controls are ensured during the year (with a strict focus on inclusion criteria, which excludes patients with acute renal disease). Yearly updates are performed to allow the inclusion of new information on patient treatment status, as well as comorbid condition updates. Detailed information on the definitions of comorbidities and coding in REIN can be found in Caillet et al. [9]. Quality controls and data collection procedures are detailed in Couchoud et al. [8].

#### The SNDS

The SNDS (a medico administrative database) collects individual data from various French health insurance schemes. This database contains exhaustive expense and reimbursement information on hospitalizations, ambulatory care, medications, laboratory analyses, and consultations for both public and private healthcare facilities, as well as transportation, compensatory daily allowances, and third-party compensatory indemnity, regardless of the payer of the services (state, complementary insurance, or out of pocket). It does not record primary care consultation diagnoses, or clinical results. For reimbursement, the SNDS includes information on long-term chronic diseases (LTD, a status that guarantees 100% reimbursement for healthcare expenses related to the disease when reported, given the fact that the patient could already been considered for LTD due to another medical condition) [4].

The HECM applied to the SNDS database uses discharge diagnoses, as well as the chronic diseases registered for healthcare reimbursement and/or specific medical acts/drugs to identify patient conditions (different algorithms for each condition, see details in supplementary Table 1). These algorithms are applied to all beneficiaries of the health insurance regimens in France (66.3 million inhabitants) that have used the healthcare system at least once during the year of interest. The pathologies, chronic treatments, and use of healthcare identified are, for the most part, non-exclusive, as the same person can be affected by several pathologies [5].

### Study population

We included patients over 18 years of age that started RRT (either dialysis or renal transplant) in France in 2018 identified through the REIN registry and who could be linked to the SNDS database.

Independently of the present study, all REIN patients were matched with SNDS patients over the available extraction period, i.e. 2006–2020 by the national coordination of REIN. with an indirect deterministic linkage that uses a combinations of 6 items: sex, age, location of residence, date and facility of kidney transplant/or start of dialysis treatment, and date of death, if available, with varying granularity (age ± 1, location at municipality or district, date ± 2 months, exact facility or facility in the same district). Further details on the linkage procedure applied yearly can be found in Raffray et al. [10]. For the purpose of this study, we selected only subjects from our incident population considered to have “good linkage”. Good linkage was defined as exact match on sex plus: either 1/ exact linkage on date of death, whatever the granularity of the other 4 items either 2/ two or more exact match on the following items: age, location of residence, date and facility of RRT. Other combinations were not included in the present study.

### Health conditions compared

For the purposes of this study, the conditions identified in the REIN registry were considered as the reference.

The following conditions identified in both the SNDS and REIN registry were included in this study: ESKD, diabetes, HIV, coronary disease, and cancer (see definition for each in Supplementary Table 1). These conditions were selected, as their identification method in both databases were comparable. In addition, the conditions studied presented an opportunity to explore the performance of the algorithms’ with different characteristics. Diabetes and HIV are disease specific and likely to be well identified in pharmaceutical records, one being very frequent, whereas the other is less. Coronary disease identification relies on mainly clinical criteria and cancer because it represents a combination of both cases. The definitions of other conditions identified with the HECM were too different compared to those in the REIN registry.

#### Statistical analysis

A descriptive analysis was performed comparing subjects with and without good REIN-SNDS linkage (patients included vs those excluded from the study). These included survival after 2018 (recruitment year), first RRT, sex, comorbid conditions, age, and regions of residence in France.

The performance of each algorithm was evaluated using sensitivity, specificity, the positive predictive value PPV), the negative predictive value (NPV), and Cohen’s kappa coefficient, together with the 95% confidence interval (CI). The level of agreement was assessed as poor (K-coefficient ≤ 0.20), fair (0.20 ≥ K-coefficient ≤ 0.40), moderate (0.40 ≥ K-coefficient ≤ 0.60), good (0.60 ≥ K-coefficient ≤ 0.80), or very good (K-coefficient ≥ 0.80) [11]. All populations included in the REIN registry had ESKD by definition. Therefore, only true positives and false negatives could be calculated for the item ESKD.

To account for the fact that HECM algorithms were designed for medico-economical purpose and individuals may not have been taken into account when they are treated at the beginning or end of the year, a secondary analysis was performed for subjects whose comorbidity data did not match for the year 2018. In these cases (unmatched conditions for 2018), the comorbidity information from the HECM for the year 2017 and 2019 were taken into consideration and new comparisons were performed. As an example, if a patient with a diabetes status did not match for the year 2018, we considered their HECM diabetes status for the year 2017 and repeated the comparison for the whole population. This secondary analysis was carried out for all conditions.

A comparison of certain characteristics was conducted (survival after 2018, first renal replacement treatment, sex, age, region of residence, nephropathy at recruitment, acute kidney disease diagnosis) to better understand the population whose conditions matched and did not match for the year 2018.

All analyses were performed using SAS enterprise guide software (version 8.3 SAS institute Inc., Cary, NC, USA).

### Ethical approval

The REIN registry creation was approved by the relevant French committees: the *Comité consultatif sur le traitement de l’information en matière de recherché* (CCTIRS N°03–149) and the *Commission nationale de l’informatique et des libertés* (CNIL N° 903,188).

The French national health insurance (CNAM) in charge of the SNDS (Système National des Données de Santé) has permanent access to the pseudonymized reimbursement data in application of the provisions of articles R. 1461‐12 et seq. of the French Public Health Code, with rules and criteria similar to the Helsinki declaration and permanent full access to the SNDS by decree (Décret n° 2016–1871 du 26 décembre 2016 relatif au traitement de données à caractère personnel dénommé « système national des données de santé»). The CNAM has authorization to perform studies based on SNDS data from the CNIL (National independent Commission for Computing and Freedom, the French data protection agency for sensitive information). All methods were carried out in accordance with relevant guidelines and regulations.

## Results

In total, 8,309 individuals were identified as incident patients in the REIN registry for the year 2018 (present in both databases). Among them, 5,971 patients were included in our study because of good linkage between the REIN and SNDS databases. The excluded population (those without good linkage) was more likely to include those who died in the year of their diagnosis, started RRT with dialysis, were older, or were a resident of Ile-de-France (Paris region) (Table 1).

**Table 1:** Description and comparison between the included and excluded populations in relationship to the linkage between the SNDS and REIN database

**Based in 2018**	Included	Excluded	Chi2
Survival beyond 2018			
Yes	5531 (93)	2097 (90)	0.001
No	440 (7)	241 (10)	
First renal replacement therapy			
Dialysis	5526 (93	2331 (100)	0.001
Transplant	445 (7)	7 (0)	
Sex			
Male	3951 (66)	1504 (64)	0.11
Female	2020 (34)	834 (36)	
Comorbid conditions			
Diabetes	2481 (42)	1219 (52)	< 0.001
HIV infection	35 (1)	22 (1)	0.13
Coronary disease	1420 (24)	624 (28)	0.0024
Cancer	622 (11)	310 (14	0.007
Age group			
00–19	93 (2)	6 (0)	0.001
20–44	586 (10)	90 (4)	
45–64	1606 (27)	557 (24)	
65–74	1558 (26)	827 (35)	
75 +	2128 (36)	858 (37)	
Region of residence			
Alsace	180 (3)	95 (4)	0.001
Aquitaine	370 (6)	98 (4)	
Auvergne	112 (2)	48 (2)	
Basse-Normandie	137 (2)	33 (1)	
Bourgogne	137 (2)	47 (2)	
Bretagne	294 (5)	85 (4)	
Centre	287 (5)	76 (3)	
Champagne-Ardenne	97 (2)	24 (1)	
Corse	8 (0)	12 (1)	
Franche-Comté	70 (1)	23 (1)	
Guadeloupe	49 (1)	16 (1)	
Guyane	17 (0)	3 (0)	
Haute-Normandie	150 (3)	75 (3)	
Ile-de-France	967 (16)	537 (23)	
Languedoc-Roussillon	359 (6)	74 (3)	
Limousin	61 (1)	10 (0)	
Lorraine	248 (4)	99 (4)	
Martinique	37 (1)	19 (1)	
Mayotte	5 (0)	8 (0)	
Midi-Pyrénées	251 (4)	79 (3)	
Nord-Pas-de-Calais	446 (8)	201 (9)	
Pays de la Loire	319 (5)	70 (3)	
Picardie	141 (2)	43 (2)	
Poitou–Charentes	117 (2)	15 (1)	
Provence-Alpes-Côte d'Azur	527 (9)	207 (9)	
Rhône-Alpes	495 (8)	252 (11)	
Réunion	90 (2)	89 (4)	

### ESKD status

With the HECM 2018 81% of the subjects with ESKD were true positives. In a secondary analysis that included information on the ESKD status from the HECM for 2019, the percentage of patients correctly identified by the SNDS database increased to 93% (Table 2). The 1,126 false negative ESKD patients (HECM 2018) were more likely died in the year they started treatment, started treatment with dialysis, among the older population, residents of Ile-de-France, and classified in the SNDS database as having acute renal disease (Table 3).

**Table 2:** Comparison between patient comorbidities in the two databases

ESKD status	TP	FN	FP	TN	Sen	Spe	PPV	NPV	Kappa
	**n (%)**	**n (%)**	**n (%)**	**n (%)**	**% (95% CI)**	**% (95% CI)**	**% (95% CI)**	**% (95% CI)**	**% (95% CI)**
REIN 2018 vs. HECM	4837 (81)	1126 (19)							
REIN 2018 vs. HECM 2017 + 2018	4837 (81)	1126 (19)							
REIN 2018 vs. HECM 2018 + 2019	5516 (93)	447 (7)							
**Diabetes**									
REIN 2018 vs. HECM 2018	2216 (37)	264 (4)	266 (4)	3182 (54)	89% (88%-91%)	92% (91%-93%)	89% (88%-91%)	92% (91%-93%)	82% (80%-83%)
REIN 2018 vs. HECM 2017 + 2018	2241 (38)	239 (4)	189 (3)	3259 (55)	90% (89%-92%)	95% (94%-95%)	92% (91%-93%)	93% (92%-94%)	85% (84%-86%)
REIN 2018 vs. HECM 2018 + 2019	2226 (38)	254 (4)	254 (4)	3194 (54)	90% (89%-91%)	93% (92%-94%)	90% (89%-91%)	93% (92%-94%)	82% (81%-84%)
**HIV**									
REIN 2018 vs. HECM 2018	29 (0.5)	6 (0.1)	15 (0.3)	5314 (99)	83% (70%-95%)	100% (100%-100%)	66% (52%-80%)	100% (100%-100%)	73% (62%-84%)
REIN 2018 vs. HECM 2017 + 2018	29 (0.5)	6 (0.1)	3 (0.1)	5326 (99)	83% (70%-95%)	100% (100%-100%)	91% (81%-100%)	100% (100%-100%)	86% (78%-95%)
REIN 2018 vs. HECM 2018 + 2019	29 (0.5)	6 (0.1)	3 (0.1)	5326 (99)	83% (70%-95%)	100% (100%-100%)	91% (81%-100%)	100% (100%-100%)	86% (78%-95%)
**Coronary disease**									
REIN 2018 vs. HECM 2018	1121 (19)	298 (5)	574 (10)	3839 (66)	79% (77%-81%)	87% (86%-88%)	66% (64%-68%)	93% (92%-94%)	62% (60%-64%)
REIN 2018 vs. HECM2017 + 2018	1169 (20)	250 (4)	292 (5)	4121 (71)	82% (80%-84%)	93% (93%-94%)	80% (78%-82%)	94% (94%-95%)	75% (73%-77%)
REIN 2018 vs. HECM2018 + 2019	1172 (20)	247 (4)	458 (8)	3955 (68)	83% (81%-84%)	90% (89%-91%)	72% (70%-74%)	94% (93%-95%)	69% (67%-71%)
**Cancer**									
REIN 2018 vs. HECM 2018	361 (7)	260 (5)	286 (6)	4057 (82)	58% (54%-62%)	94% (93%-95%)	56% (52%-60%)	95% (94%-95%)	51% (48%-55%)
REIN 2018 vs. HECM 2017 + 2018	391 (7)	230 (4)	235 (4)	4576 (84)	63% (59%-67%)	95% (95%-96%)	62% (59%-66%)	95% (95%-96%)	57% (54%-61%)
REIN 2018 vs. HECM 2018 + 2019	386 (7)	235 (4)	245 (4)	4570 (84)	62% (58%-66%)	95% (94%-96%)	62% (58%-65%)	95% (95%-96%)	57% (53%-60%)

**Table 3:** Characteristics of the matched and unmatched populations by disease

Based in 2018 conditions										
	**ESKD**		**Diabetes**		**HIV**		**Coronary disease**		**Cancer**	
**Survival beyond 2018**	**Matched**	**Not Matched**	**Matched**	**Not Matched**	**Matched**	**Not Matched**	**Matched**	**Not Matched**	**Matched**	Not Matched
**Yes**	4567 (94)	964 (86)	5044 (93)	487 (92)	5511 (93)	20 (95)	4742 (93)	789 (90)	4528 (93)	486 (89)
**No**	278 (6)	162 (14)	397 (7)	43 (8)	439 (7)	1 (5)	357 (7)	83 (10)	358 (7)	60 (11)
**Treatment at recruitment**										
** Dialysis**	4415 (91)	1111 (99)	5049 (93)	477 (90)	5505 (93)	21 (100)	4692 (92)	834 (96)	4886 (100)	546 (100)
** Transplant**	430 (9)	15 (1)	392 (7)	53 (10)	5505 (7)	0 (0)	407 (8)	38 (4)	0 (0)	(0)
**Sex**										
** Male**	3183 (66)	768 (68)	3577 (66)	374 (71)	3939 (66)	12 (57)	3326 (65)	625 (72)	3189 (65)	420 (77)
** Female**	1662 (34)	358 (32)	1864 (34)	156 (29)	2011 (34)	9 (43)	1773 (35)	247 (28)	1697 (35)	126 (23)
**Age group**										
** 00–19**	85 (2)	8 (1)	92 (2)	1 (0)	93 (2)	0 (0)	93 (2)	0 (0)	64 (1)	2 (0)
** 20–44**	521 (11)	65 (6)	565 (10)	21 (4)	585 (10)	1 (5)	553 (11)	33 (4)	443 (9)	8 (1)
** 45–64**	= !1372 (28)	234 (21)	1464 (27)	142 (27)	1598 (27)	8 (38)	1448 (28)	158 (18)	1329 (27)	65 (12)
** 65–74**	1209 (25)	349 (34)	1389 (26)	169 (32)	1553 (26)	5 (24)	1270 (25)	288 (33)	1297 (27)	178 (33)
** 75 + **	1658 (34)	470 (42)	1931 (35)	197 (37)	2121 (36)	7 (33)	1735 (34)	393 (45)	1753 (36)	293 (54)
**Region of residence**										
** Alsace**	138 (3)	42 (4)	159 (3)	21 (4)	178 (3)	2 (10)	141 (3)	39 (4)	140 (3)	25 (5)
** Aquitaine**	307 (6)	63 (6)	342 (6)	28 (5)	369 (6)	1 (5)	318 (6)	52 (6)	312 (6)	27 (5)
** Auvergne**	88 (2)	24 (2)	105 (2)	7 (1)	112 (2)	0 (0)	95 (2)	17 (2)	97 (2)	11 (2)
** Basse-Normandie**	116 (2)	21 (2)	133 (2)	4 (1)	137 (2)	0 (0)	115 (2)	22 (3)	113 (2)	14 (3)
** Bourgogne**	109 (2)	28 (2)	129 (2)	8 (2)	136 (2)	1 (5)	121 (2)	16 (2)	123 (2)	6 (1)
** Bretagne**	245 (5)	49 (4)	274 (5)	20 (4)	293 (5)	0 (0)	262 (5)	32 (4)	243 (5)	24 (4)
** Centre**	231 (5)	56 (5)	270 (5)	17 (3)	287 (5)	0 (0)	227 (4)	60 (7)	227 (5)	41 (8)
** Champagne-Ardenne**	81 (2)	16 (1)	87 (2)	10 (2)	97 (2)	0 (0)	79 (2)	18 (2)	90 (2)	3 (1)
** Corse**	8 (0)	0 (0)	8 (0)	0 (0)	8 (0)	0 (0)	7 (0)	1 (0)	6 (0)	2 (0)
** Franche-Comté**	61 (1)	9 (1)	65 (1)	5 (1)	70 (1)	0 (0)	54 (1)	16 (2)	54 (1)	7 (1)
** Guadeloupe**	42 (1)	7 (1)	44 (1)	5 (1)	49 (1)	0 (0)	44 (1)	5 (1)	40 (1)	5 (1)
** Guyane**	13 (0)	4 (0)	15 (0)	2 (0)	16 (0)	1 (5)	16 (0)	1 (0)	16 (0)	1 (0)
** Haute-Normandie**	134 (3)	16 (1)	135 (2)	15 (3)	150 (3)	0 (0)	132 (3)	18 (2)	114 (2)	13 (2)
** Ile-de-France**	747 (15)	220 (20)	832 (17)	135 (25)	961 (16)	6 (29)	827 (16)	140 (16)	761 (16)	95 (17)
** Languedoc-Roussillon**	285 (6)	74 (7)	326 (6)	33 (6)	359 (6)	0 (0)	307 (6)	52 (6)	220 (5)	30 (5)
** Limousin**	50 (1)	11 (1)	58 (1)	3 (1)	61 (1)	0 (0)	57 (1)	4 (0)	52 (1)	8 (2)
** Lorraine**	205 (4)	43 (4)	237 (4)	11 (2)	248 (4)	0 (0)	212 (4)	36 (4)	220 (5)	17 (3)
** Martinique**	26 (1)	11 (1)	34 (1)	3 (1)	37 (1)	0 (0)	35 (1)	2 (0)	33 (1)	1 (0)
** Mayotte**	4 (0)	1 (0)	4 (0)	1 (0)	5 (0)	0 (0)	4 (0)	1 (0)	4 (0)	0 (0)
** Midi-Pyrénées**	206 (4)	45 (4)	233 (4)	18 (3)	251 (4)	0 (0)	215 (4)	36 (4)	200 (4)	19 (3)
** Nord-Pas-de-Calais**	357 (7)	89 (8)	405 (7)	41 (8)	445 (7)	1 (5)	373 (7)	73 (8)	387 (8)	44 (8)
** Pays de la Loire**	279 (5)	63 (6)	300 (6)	19 (4)	319 (5)	0 (0)	281 (6)	38 (4)	254 (5)	34 (6)
** Picardie**	116 (2)	25 (2)	130 (2)	11 (2)	141 (2)	0 (0)	124 (2)	17 (2)	124 (3)	9 (2)
** Poitou–Charentes**	102 (2)	15 (1)	110 (2)	7 (1)	117 (2)	0 (0)	106 (2)	11 (1)	86 (2)	14 (3)
** Provence-Alpes-Côte d'Azur**	439 (9)	88 (8)	478 (9)	49 (9)	520 (9)	7 (33)	443 (9)	84 (10)	426 (9)	52 (10)
** Rhône-Alpes**	408 (8)	87 (8)	451 (9)	44 (8)	494 (8)	1 (5)	426 (8)	69 (8)	380 (8)	40 (7)
** Reunion**	71 (1)	19 (2)	77 (1)	13 (2)	90 (2)	0 (0)	78 (2)	12 (1)	76 (2)	4 (1)
**Nephropathy at recruitment**										
** Polycystic renal disease**	331 (7)	46 (4)	349 (6)	28 (5)	377 (6)	0 (0)	338 (7)	39 (4)	279 (6)	13 (2)
** Hypertension**	1171 (24)	297 (26)	1323 (24)	409 (26)	1463 (25)	5 (24)	1226 (24)	242 (28)	1275 (26)	160 (30)
** Unknown**	740 (15)	193 (17)	851 (16)	244 (16)	929 (16)	4 (19)	761 (15)	172 (20)	780 (16)	102 (19)
** Other**	767 (16)	174 (15)	847 (16)	227 (15)	939 (16)	2 (9.62)	842 (17)	99 (11)	712 (15)	102 (17)
** Diabetic nephropathy**	922 (19)	261 (23)	1089 (20)	372 (24)	1181 (20)	2 (10)	963 (19)	228 (26)	1042 (20)	84 (15)
** Primary glomerulonephritis**	601 (12)	94 (8)	634 (12)	144 (9)	691 (12)	4 (19)	631 (12)	64 (7)	567 (12)	38 (7)
** Pyelonephritis**	234 (5)	52 (5)	266 (5)	67 (4)	282 (5)	4 (19)	264 (5)	22 (3)	196 (4)	40 (7)
** Vascular Disease**	49 (1)	9 (1)	55 (1)	12 (1)	58 (1)	0 (0)	47 (1)	11 (1)	35 (1)	7 (1)
**Acute kidney disease (SNDS)**										
** No**	4837 (100)	423 (48)	4785 (88)	475 (90)	5239 (88)	21 (100)	4489 (88)	771 (88)	4256 (87)	493 (90)
** Yes**	0 (0)	703 (62)	648 (12)	55 (10)	709 (12)	0 (0)	602 (12)	101 (12)	636 (13)	53 (8)

### Diabetes

Forty-two percent of the population identified in the REIN database were registered as having diabetes. Eight percent of the population’s diabetes identification differed between the databases (distributed equally between false positives and false negatives) for their diabetes status between the two databases for HCEM 2018 (Table 2). The population of 530 patients with differing diabetes status had a higher proportion of patients who had transplantation as their first RRT, were over 75 years of age, or were residents of Ile-de-France (Table 3). The Kappa coefficient of agreement was found to be very good (82%), as were the specificity, sensitivity, NPV, and PPV (over 89%). No great improvement was observed when including the patients’ diabetes status in the HECM for 2017 or 2019.

### HIV infection

Only 1% of patients identified in the REIN database were HIV positive. Approximately 0.4% of the population differed between the databases based on their HIV/AIDS status (Table 2). Among the 21 disparate patients based on HIV status, no transplant patients were misclassified, a higher percentage were aged between 45 and 64, and most were identified as residents of Ile-de-France (Table 3). This comparison showed a good Kappa coefficient of agreement. The sensitivity and PPV were the lowest among the other parameters measured, with 83% and 66%, respectively. An improvement to 0.2% was observed for the false positives when including information from the HECM the year before and after recruitment.

### Coronary disease

Twenty four percent of the patients identified in the REIN database were recorded as having coronary disease. Fifteen percent of the population differed on coronary disease status, of which two thirds of the disparate patients were false positives (Table 2). The 872 unmatched patients based on coronary disease status were more likely to be patients who died early or started treatment with dialysis (Table 3). The sensitivity was 79% and specificity 87%. The Kappa coefficient of agreement between the REIN and SNDS databases on coronary disease was 62%. The level of agreement improved to 75% and 69% when considering the information from 2017 and 2019 from the HECM, respectively.

### Cancer

Eleven percent of the population was identified as having cancer. Cancer status differed in 10% of the patients (Table 2). The 546 unalike patients based on cancer status died early, all started treatment with dialysis, and were more likely to be part of the older group (Table 3). Sensitivity was 58% and specificity 94%. This comparison showed the lowest PPV of the comorbidities studied, with 56%, as well as only a 51% kappa coefficient of agreement.

## Discussion

In this study, we compared the information on patient conditions between the REIN registry collected based on clinical data and the HECM algorithm based on health consumption reimbursement data. The agreement between diagnoses as identified by the REIN and the SNDS varied between conditions, with the highest for diabetes and the lowest for cancer. Specificity was above 85% and the PPV over 95% for all conditions, suggesting overall good performance of the HECM algorithms in identifying the conditions of interest in this study.

### Ease of diagnosis

Pathologies with tracer drugs or tracer medical acts are better identified in medico-administrative refund information databases [12, 13]. The Kappa coefficients for the status of diabetes and HIV were higher than those for coronary disease and cancer. These comorbidities identified in the SNDS database are treated with medications that are specifically used for the disease, allowing us to identify patients whose LTD registration or hospitalization diagnoses are not reported. Coronary disease as a medical diagnosis is slightly more difficult to identify in the SNDS database, as it relies on discharge records for patients hospitalized during the given period or an LTD reported in the four years before the year of interest. There are no specific drugs or medical procedures that are integrated into the HECM that can help identify patients who do not comply with the specified conditions. The REIN database benefits from direct patient interviews and medical records to record information on these conditions.

The definition of active cancer in the HECM is based on patients with a reported LTD and hospital diagnosis during the year. These definitions could lead to an underestimation of patients who either did not receive treatment or whose treatment was received in ambulatory care (whose LTD is not reported for the year of interest). As an example, a patient receiving antiestrogen therapy for breast cancer treatment in an ambulatory setting, without hospitalization associated with the reported disease and no LTD reported could be missed by the HECM tool [14]. On the contrary, the REIN database reports active cancer regardless of the patients’ current treatment status. These differences in definition could explain some of the false negative patients.

The sensitivity and specificity were high (> 80) for most of the assessed diseases, except the sensitivity for coronary disease. This high level of sensitivity suggests that the HECM tool is able identify patients with a disease (unlikely to produce false negatives). High specificity was seen for all comorbidities assessed, suggesting a low number of patients being categorized as having the condition when they do not (false positives).

### Timelines

In comparing these databases, we should consider that the REIN database collects information prospectively and that the HECM categorizes diseases retrospectively for a given year. The identification of patients with ESKD improved when adding information from the year 2019. A great number of patients with mismatched ESKD status were found to be patients coded in the SNDS as acute kidney disease in the REIN incident year. These may have been patients with chronic kidney disease but who started chronic dialysis after an acute episode who did not fulfill the required time under treatment to be classified as ESKD by the end of the year of interest. As well, despite the work of the REIN registry's research assistants, whose mission is to check the completeness of the cases and compliance with the protocol, we cannot rule out a few marginal errors.

Concerning false negatives for diabetes, a patient identified in the REIN database in December as being diabetic that did not fulfill the requirements to be identified by HECM (e.g., needs 3 antidiabetic drug deliveries to be identified through medication) for that year would have resulted in a mismatch. A patient identified in the REIN database in January as a patient without cancer might have developed the disease later in the same year and the HECM tool would register them as positive for cancer in that same year, resulting in a false positive. For, coronary disease, we observed better performance when data for the year 2017 was added. This could be a result of HECM considering data for the four years prior to the year of interest to classify patients, therefore, including adding information for patients from 2015.

### Patient characteristics

We explored the characteristics between the unmatched and matched populations for each condition. We found a higher proportion of early deaths, first RRT with dialysis, males, and residents of Ile-de-France among the unmatched population. Patients with short survival would not have the opportunity to have their record corrected in the REIN database and in the SNDS, they may not have had sufficient healthcare consumption to be identified. First RRT treatment with dialysis and residency in Ile-de-France were the biggest subgroups for which linkage was more likely to be less precise. The Ile-de-France region is a densely populated region were patients could easily mobilize between the different facilities [8]. Patients could start their treatment at an ICU (recorded in the SNDS) in a postal code and later transferred to a less medicalized center elsewhere (recorded in the REIN database). The prevalence of a disease in a population can influence the PPV and NPV. When prevalence increases the PPV increases but the NPV decreases [15, 16]. In this population, the prevalence of diabetes, coronary disease, and cancer was higher than in the general population (prevalence estimated to be 5.88%, 3.11%, and 4.98% in 2018, respectively, for the general French population [17]). These accuracy parameters (PPV and NPV) may, therefore, not be replicable in the general population.

### Strengths and limitations

The strengths of this study were that it used two national databases in which comorbidities are identified by two different methods. However, this study also had several limitations. First, even though the parameters to categorize a patient as having or not a condition are comparable between both databases they are not identical. Therefore, certain patients’ conditions could be disparate eeven when correctly categorized in both databases. Unfortunately, among the 58 conditions of the HECM, only 5 had similar identification method with REIN. Many medical conditions explored by the HECM are not collected in REIN like precise cancer location or psychiatric disorder or neurodegenerative disease. We recognise that the results observed for these 5 diseases would have been significantly poorer if we had used diseases whose identification method initially differed.

Second, for legal reasons the databases used do not have a shared unique identifier for patients and therefore relied on a direct deterministic algorithm to link patient information between them. Even when only including patients with a good linkage, there might have been certain patients who were imperfectly linked. The choice to keep only patients with a good match led to the exclusion of 2,338 patients. It seemed to us that in the case of our objective, this did not constitute a bias but may reduced the scope of the extrapolation of our comparison.

Third, HECM algorithms were designed for medico-economic rather than epidemiological purposes. As such, they do not aim to collect the exhaustive number of incident cases over one year, as economists are generally more interested in the longitudinal evolution of healthcare expenditure and consumption, observed on specific samples. The pathologies categorized by the algorithm are based on short periods, with individuals not taken into account when they are treated at the beginning or end of the year. This may explain the improvement in performance when the search was extended to the years 2017 and 2019. On the other hand, despite the fact that completeness and accuracy are ascertained by REIN research assistants during regular visits in every dialysis centre, and update at each annual visit, we may not exclude coding error in transcription from medical record*.*

The REIN database included only patients with ESKD, representing only a small proportion of the French population. Therefore, the generalisability of the results to other populations should be explored. Other French registries have successfully linked most of their patients (all over 85%) to the SNDS database: CONSTANCES, FRESH HR, ACIRA, France-TAVI, CANARI [18–21]. These linkages have been used to enrich the databases of the registries and could potentially be used as a starting point to further validate the HECM tool.

## Conclusion

The development of tools that allow the use of medico-administrative databases for epidemiological research is of great important, as they provide information at the national level, limiting the costs and time required for more traditional data collection methods. The HECM algorithm matched the information provided by the REIN database with that of the SNDS database relatively well. However, further validation of the HECM tool on other populations should be performed.

### Supplementary Information

**Additional file 1:** **Supplementary Table 1.** Comparison between the definitions used in the REIN and SNDS databases.

## Data Availability

The author is authorized by the REIN scientific council to use the datasets used and/or analysed during the current study. These databases are available from the corresponding author on reasonable request.
