# Supplementary material for: Cross-validation of comorbidity items in two national databases in a sample of patients with end-stage kidney disease
Source: BMC Health Serv Res. 2023 Oct 24;23:1140. doi: 10.1186/s12913-023-10145-y (PMC10594771; doi:10.1186/s12913-023-10145-y)
Supplement: Supplementary file 1 — Additional file 1: Supplementary Table 1. Comparison between the definitions used in the REIN and SNDS databases. [file 12913_2023_10145_MOESM1_ESM.docx]

| **Supplementary Table 1. Comparison between the definitions used in the REIN and SNDS databases** | |
| --- | --- |
| **Chronic kidney disease** | |
| **REIN** | - Concerns all patients undergoing replacement therapy for chronic renal failure by dialysis or renal transplantation in metropolitan France and in the French overseas territories. - A patient is considered to be in chronic renal failure if he/she receives dialysis for more than 45 days or pre-emptive transplantation. - In case of death before the 45th day, an expert opinion will be consulted to differentiate between chronic renal failure and acute renal failure. |
| **SNDS** | **Chronic dialysis:**   - People with at least 45 days of hemodialysis or at least one day of peritoneal dialysis in year n. - People with at least one day of hemodialysis (and less than 45 days) in year n and at least 45 days of hemodialysis or at least one day of peritoneal dialysis in year n-1.   **Renal transplantation:**   - People identified by a GHM (Groupe Homogène de Malades) for renal transplantation (regardless of the level of severity). - People identified by a CCAM (Classification Commune des Actes Médicaux) procedure for kidney transplantation or pancreas and kidney transplantation, by laparotomy. Kidney transplantation in year n.   **Renal transplant follow up:**   - People who received at least three deliveries (at different dates) of at least one immunosuppressive drug used against kidney transplant rejection and listed on the reimbursable drug list. - People hospitalized for kidney transplant monitoring (regardless of the level of severity) during years n to n-4 - People hospitalized with a diagnosis of kidney transplantation during years n to n-4. - People identified by a GHM or a CCAM act of kidney transplantation or of pancreas and kidney transplantation for years n-1 to n-4. - Having an LTD (affection de longue durée) during year n for chronic renal insufficiency.   Kidney transplant follow-up takes precedence over chronic dialysis and kidney transplantation takes precedence over kidney transplant follow-up. |
| **Diabetes** | |
| **REIN** | - Patients with a fasting blood glucose ≥ 7.0 mmol/l (1.26 g/l) or postprandial blood glucose ≥ 11.1 mmol/l (2 g/l) on two occasions. - Type 1 or 2 diabetes - Patient treated with insulin. |
| **SNDS** | - People who received at least three deliveries (on different dates) of oral antidiabetic drugs or insulin (or at least 2 in case of 1 large package) during year n. - People who received at least three deliveries (on different dates) of oral antidiabetic drugs or insulin (or at least 2 in case of at least 1 large package) during year n-1. - People with an LTD in year n with ICD-10 codes for diabetes and/or people hospitalized in years n to n-1 with ICD10 codes for diabetes. People hospitalized in years n to n-1 for a complication of diabetes (PD or RD) with an ICD10 code for diabetes as a diagnosis. |
| **Coronary insufficiency** | |
| **REIN** | History of angioplasty or bypass surgery or coronary artery disease documented by stress ECG, coronary angiography, or thallium scintigraphy. |
| **SNDS** | Grouping of acute coronary syndrome and chronic coronary disease conditions:  - People hospitalized in year n for acute ischemic heart disease (principal diagnosis (PD) of one of the RUMs (resumé d’unité médicale - summary of the medical unit).  - People with an LTD during year n with ICD-10 codes for ischemic heart disease.  - People hospitalized during years n to n-4 for these same reasons.  - People hospitalized in year n for any other reason with ischemic heart disease as an associated complication or morbidity. |
|  | **HIV infection** |
| **REIN** | - Patients falling into Groups 1 (acute infection) or 2 (asymptomatic patient) or III (persistent generalized   lymphadenopathy) of the Center for Disease Control (CDC) classification.   - AIDS Patients falling into group 4 (ARC, opportunistic infections, secondary tumors, neurological disease etc.) of the CDC classification. |
| **SNDS** | - People with an LTD during year n with ICD-10 codes for diseases due to human immunodeficiency virus. - People hospitalized in MCO (médecine, chirurgie, obstétrique – medicine, surgery, obstetrics) under this diagnosis or for the same reasons during years n to n-4. - People hospitalized in MCO during year n for any other reason with HIV/AIDS as a complication or associated morbidity - People who had a delivery on three different dates of at least one drug specific to the treatment of HIV/AIDS during year n. - People who had a medical biology procedure specific to the treatment of HIV/AIDS during year n. |
| **Cancer** | |
| **REIN** | Patients with cancer or hematological malignancy not considered to be in remission, especially if undergoing treatment (chemotherapy, radiotherapy, surgery, or palliative care) or with metastases. |
| **SNDS** | Grouping of pathologies: active female breast cancer, active colon cancer, active lung cancer, active prostate cancer, and other active cancers.  **Breast Cancer:**   - Women with an LTD with ICD-10 codes for breast cancer with a date of onset in years n to n-1. - Women hospitalized for breast cancer in years n to n-1 (principal diagnosis (PD) or related diagnosis (RD)). Active cancer takes precedence over cancer under surveillance.   **Colon cancer:**   - People with an LTD with ICD-10 codes for cancer of the colon, recto-sigmoid junction, or rectum (including in situ forms), with a date of onset in years n to n-1. - People hospitalized for cancer of the colon, recto-sigmoid junction, or rectum (including in situ forms) in years n to n-1 (principal diagnosis (PD) or related diagnosis (DR)).   **Lung cancer:**   - People with an LTD with ICD-10 codes for lung or bronchial cancer (including in situ forms) with date of onset in years n to n-1. - People hospitalized for lung or bronchial cancer in years n to n-1 (principal diagnosis (PD) or related diagnosis (DR)).   **Prostate:**   - Men hospitalized with prostate cancer in years n to n-1 (principal diagnosis (PD) or related diagnosis (RD)). - Men aged 40 years and older who received at least three deliveries in years n to n-1 of anti-androgenic treatment.   **Other Cancers**   - People with an LTD with ICD-10 codes for other cancers (excluding breast (in women), colon, recto-sigmoid junction, rectum, lung, bronchus, and prostate cancer and tumors with unpredictable or unknown course) with onset in years n to n-1. - People hospitalized with other cancers in years n to n-1 (principal diagnosis (PD) or related diagnosis (RD)), and men with breast cancer. |
